# Automated Estimation of Acute Infarct Volume from Noncontrast Head CT Using Image Intensity Inhomogeneity Correction

**DOI:** 10.1155/2019/1720270

**Published:** 2019-08-21

**Authors:** Keith A. Cauley, Gino J. Mongelluzzo, Samuel W. Fielden

**Affiliations:** ^1^Department of Radiology, Geisinger, Danville, PA 17821, USA; ^2^Department of Imaging Science & Innovation, Geisinger, Danville, PA 17821, USA; ^3^Department of Medical & Health Physics, Geisinger, Danville, PA 17837, USA

## Abstract

Identification of early ischemic changes (EIC) on noncontrast head CT scans performed within the first few hours of stroke onset may have important implications for subsequent treatment, though early stroke is poorly delimited on these studies. Lack of sharp lesion boundary delineation in early infarcts precludes manual volume measures, as well as measures using edge-detection or region-filling algorithms. We wished to test a hypothesis that image intensity inhomogeneity correction may provide a sensitive method for identifying the subtle regional hypodensity which is characteristic of early ischemic infarcts. A digital image analysis algorithm was developed using image intensity inhomogeneity correction (IIC) and intensity thresholding. Two different IIC algorithms (FSL and ITK) were compared. The method was evaluated using simulated infarcts and clinical cases. For synthetic infarcts, measured infarct volumes demonstrated strong correlation to the true lesion volume (for 20% decreased density “infarcts,” Pearson r = 0.998 for both algorithms); both algorithms demonstrated improved accuracy with increasing lesion size and decreasing lesion density. In clinical cases (41 acute infarcts in 30 patients), calculated infarct volumes using FSL IIC correlated with the ASPECTS scores (Pearson r = 0.680) and the admission NIHSS (Pearson r = 0.544). Calculated infarct volumes were highly correlated with the clinical decision to treat with IV-tPA. Image intensity inhomogeneity correction, when applied to noncontrast head CT, provides a tool for image analysis to aid in detection of EIC, as well as to evaluate and guide improvements in scan quality for optimal detection of EIC.

## 1. Introduction

Noncontrast head CT (NCCT) is typically first-line in stroke evaluation and is used to identify acute hemorrhage as a contraindication for IV-tPA. It is also used to estimate the size and age of the stroke, as IV-tPA or intra-arterial thrombectomy may be contraindicated for larger [1–5] and more mature strokes [6–8]. Infarct size also correlates with long-term functional outcome [9, 10]. Very early stroke can be difficult to discern on NCCT, and a number of criteria have been published regarding early NCCT signs of acute stroke [11–13], with studies showing limited consensus among experienced readers [14, 15].

Here we propose a novel application of image intensity inhomogeneity correction (IIC) to identify and quantify acute infarct volume. IIC is a broadly-applied image preprocessing technique designed to remove subtle, smoothly varying intensity shading caused by system nonidealities. We test a hypothesis that publicly available IIC algorithms, along with simple Hounsfield Unit (HU) thresholding, will be sensitive in detecting small intensity abnormalities caused by stroke and that stroke volumes may be estimated from the resulting intensity correction maps.

## 2. Materials and Methods

### 2.1. Simulated Infarcts

To investigate the ability of the tool to identify known volumes of decreased radiodensity, areas of decreased radiodensity were created using FSL tools and a control NC head CT. Synthetic infarcts were created with a range of decreased radiodensities approximating the middle cerebral artery (MCA) territory. Additionally, spherical hypodensities of different sizes within this territory were generated. Spherical volumes were used to maintain consistency of shape with changes in volume [16]. The resulting images were passed through the processing pipeline described above. This simulation study was not designed to reflect specifics regarding vascular territories or ASPECTS areas.

### 2.2. Imaging Data

This retrospective study was approved by the IRB of our institution. Standard clinical CT scanners with helical acquisition and head CT protocol with 135kVp and modulated MA, min 50 and max 290 mA, rotation time 0.75s were used for NCCT performed from the foramen magnum through the vertex with standard 512x512 matrix, 24- cm field of view at 5.0-mm section thickness. In some cases images were reoriented after acquisition at the console or using Mango software (Research Imaging Institute, UTHSCSA). Routine follow-up MRI was performed, in most cases within 24 hrs.

DICOM images were converted to the NIfTI (Neuroimaging Informatics Technology Initiative) data format using MRIConvert-2.0.7 (http://lcni.uoregon.edu/~jolinda/MRIConvert/). The data that support the findings of this study are available from the corresponding author, KAC, upon reasonable request.

### 2.3. Image Processing

**Figure 1:**
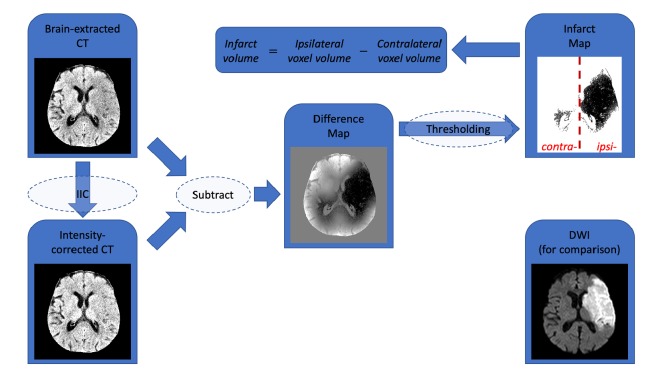
Illustration of the image processing pipeline. After brain-extraction, the NCCT is passed through IIC to generate the intensity-corrected or “restored” image. The input image is then subtracted from the product image to generate a difference map highlighting areas of reduced radiodensity. After thresholding, the volume of the contralateral hemisphere difference map is subtracted from volume of the ipsilateral hemisphere difference map to yield the infarct volume. An MR diffusion-weighted image of the same case performed the following day is included for reference.

Images were thresholded at 50HU to remove skull and bone, and brain was extracted as previously described [17]. As the novel part of this pipeline, IIC data, and therefore difference maps, were obtained from two publicly-available algorithms: FSL automated segmentation tool (FAST), imbedded within FSL, which estimates image intensity inhomogeneity as part of a segmentation algorithm [18], and N4ITK, which does not concurrently perform tissue segmentation [19]. For brevity, these algorithms will be referred to as FSL and ITK for the remainder of this manuscript. In the case of FSL, brain extracted images were segmented into 2 compartments using FAST. FAST uses IIC in its segmentation algorithm and has the option of reporting a “restored” input image (FSL nomenclature), in which slowly-varying intensity nonuniformities have been removed. By subtracting this “restored” image from the input image, a “difference map” can be obtained. This difference map shows the absolute HU correction and includes both baseline corrections from the “cupping” artifacts and any other intensity inhomogeneities [20], as well as the infarct. ITK does not involve segmentation but will generate a similar “restored” image which can be used to generate a difference map. In each case the map was divided into ipsilateral and contralateral hemispheres using the fslroi tool, and they were thresholded at an upper value of -1.5HU to minimize the effects of inhomogeneity artifacts. The thresholded contralateral hemisphere map shows baseline inhomogeneities caused by various CT artifacts, and the ipsilateral map shows the infarct as well as the additional inhomogeneities. Subtracting the volume of the contralateral correction from the volume of the ipsilateral correction serves to normalize for baseline image intensity inhomogeneities and permits estimation of the infarct volume. The image processing pipeline is shown in Figure 1.

In some cases (15 of 41) acute infarct boundaries could be estimated and infarct volumes were estimated manually. Volumes were estimated at the clinical work station using a freehand ROI tool with ROI drawn on axial images. Final infarct volumes were measured from CT or MRI diffusion weighted images.

### 2.4. Subjects

#### 2.4.1. Stroke Patients

A query of the neuroradiology department database for acute infarct at our institution was conducted. Inclusion criteria were acute MCA stroke with NCCT with stroke verified by subsequent imaging. ICA/MCA strokes were included. Exclusion criteria were hemorrhage, previous infarct, bilateral hemispheric infarct, very small or embolic infarcts, previous craniotomy, or metal in the field of imaging. Of 550 stroke alerts from January to September 2017, 30 patients (19M, 11F, average age 69) and 41 head CTs met inclusion criteria (see Supplementary Data Table for summary of patient demographics (available here)). NIHSS scores and information regarding the use of IV-tPA were obtained from the clinical notes. NIHSS was recorded for 22 of the 30 patients.

ASPECTS scoring: two neuroradiologists familiar with ASPECTS scoring independently reviewed all studies in double blind fashion to generate an ASPECTS score. The radiologists performed their scoring on axial NCCT images at a PACs workstation and were permitted to adjust the window setting as needed but were not permitted to use a radiodensity measuring tool (ROI). The final reported ASPECTS score is an average score.

#### 2.4.2. Statistical Methods

Graphpad PRISM was used for Student's unpaired t-tests and Pearson correlations.

## 3. Results

### 3.1. Simulated Infarcts

**Figure 2:**
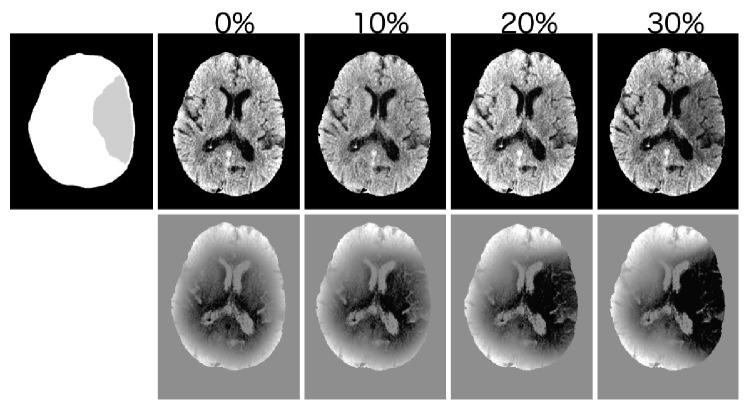
Simulated MCA territory infarcts. The figure illustrates the visual appearance of simulated MCA-territory infarcts of various (known) attenuations (top) and the appearance of each known attenuation on the difference map (bottom). The simulated territory is shown top-left for reference. Infarcts of less than 20% decreased density show poor margin delineation.

The image processing pipeline was tested using “synthetic infarcts” as an area of decreased radiodensity where the size and radiodensity of the infarct are known.  Figure 2 shows that infarcts resulting in 10% decrease in radiodensity (approximately 3HU) are not visually evident but can be identified as an image intensity discrepancy when compared to the contralateral hemisphere. The hypodensity becomes visually apparent at 20% density decrease and demarcated at 30% decrease in density. The corresponding IIC difference map is seen below each figure.

**Figure 3:**
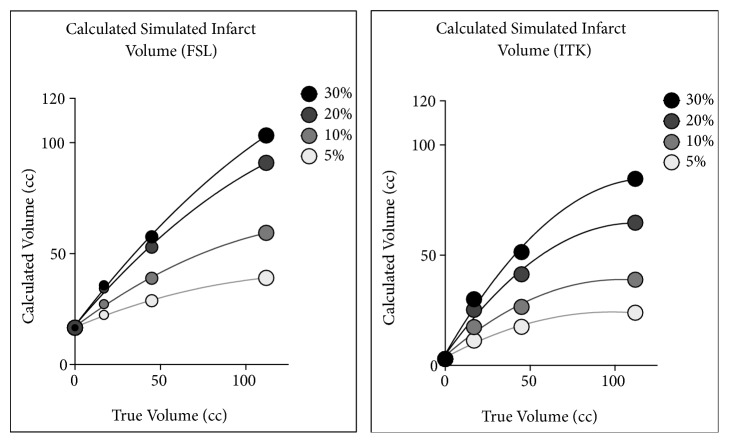
Calculated infarct volume plotted against true infarct volume, for (3) different input volumes (122cc, 45cc, and 17cc) and (4) different infarct densities (with density reduced by 5%, 10%, 20%, and 30%), FSL (left), ITK (right).

Synthetic spherical hypodensities served to standardize the shape and permitted a more predictable hypodensity volume, as has been done previously [16]. Three different spherical volumes (112, 45, and 17cc) each of 4 different radiodensities (5%, 10%, 20%, and 30% decrease in radiodensity) were investigated. Spherical hypodensities were digitally introduced into the right MCA territory of a control (normal) head CT. In this way the calculated infarct volume and radiodensity could be compared with the “true” infarct volume and radiodensity. Results are summarized in Figure 3 and Table 1. In all cases, the infarct volume calculated using ITK was smaller than that calculated from FSL.

### 3.2. Clinical Cases

**Figure 4:**
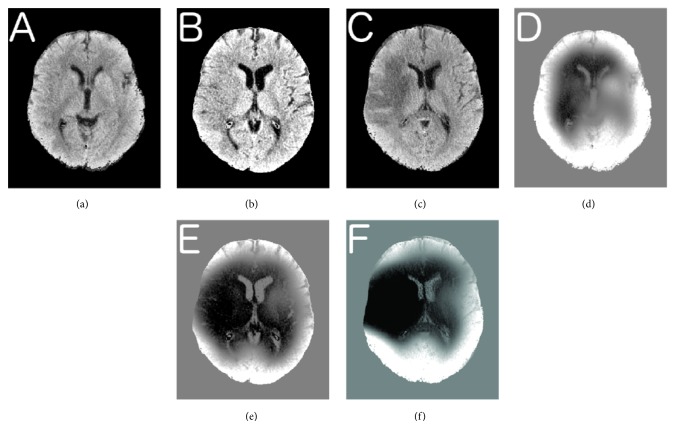
Time series images. Initial brain extracted image at 2.5 hours after symptom onset (a), at 5 hrs (b), and at 28 hrs (c). Corresponding thresholded difference maps (d–f). Stroke volumes were estimated (FSL) 12cc, 81.4cc, and 117.9cc as the infarct evolved.

Noncontrast head CTs from 30 acute stroke patients, 41 noncontrast CT studies, were processed using the image analysis pipeline outlined in Figure 1 and example case illustrated in Figure 4. IIC algorithms from FSL and ITK were compared with outcome measure as correlation with admission NIHSS, ASPECTS score, manual volume estimates, and decision to treat with IV-tPA.

### 3.3. Measured Infarct Volume Correlated with Manual Infarct Measures

Hyperacute infarcts are poorly delineated and not amenable to manual volume estimates. However our study includes 14 more mature infarcts, where manual estimates were possible. In these cases, manual volume estimates showed strong correlation with IIC measurements using FSL, Pearson's r = 0.823, p=0.0002.

### 3.4. Measured Infarct Volume Correlated with the Admission NIHSS

For the 22 patients with recorded admission NIHSS, the measured infarct volume was correlated with the NIHSS. For FSL, Pearson's r = 0.544, p < 0.05 (strong correlation Pearson's r > 0.5). Though the two IIC methods correlated with each other (Pearson's r = 0.766) the correlation of the ITK-based algorithm with the NIHSS was poor, with Pearson's r = 0.238.

### 3.5. Measured Infarct Volume Correlated with ASPECTS Scores

**Figure 5:**
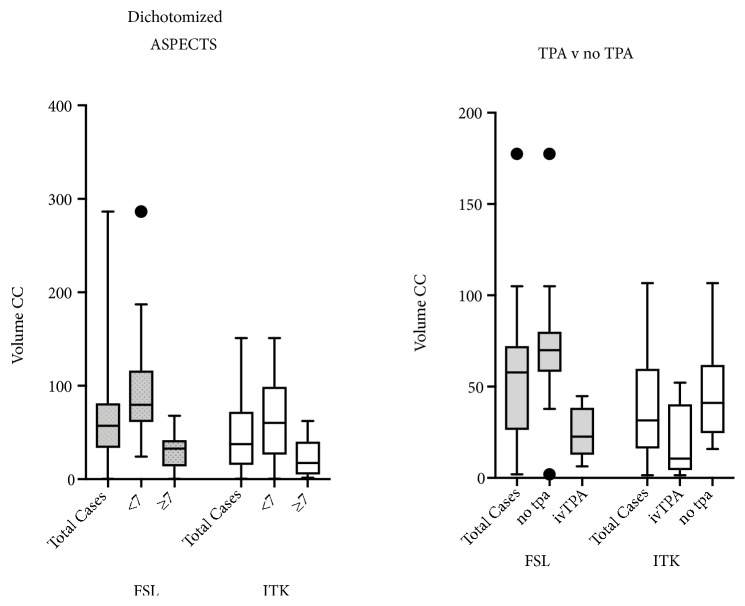
Box and whiskers plot of calculated stroke volumes as a function of the IIC program used (FSL or ITK), correlated with dichotomized ASPECTS scores (left) and clinical decision to treat with IV TPA (right).

Measured infarct volumes were correlated with ASPECTS scores. For all cases (41 cases), for FSL Pearson's, r =0.68. ASPECTS scores were also dichotomized at ≥7 and <7. For ≥7 (n=16) mean volume = 28.8cc, Std deviation 58.38cc. For <7 (n=24) mean volume = 94.2cc, SD 19cc. The two groups were significantly different, Students t-test p < 0.0001. For ITK, Pearson's r = 0.68 for all cases. For dichotomized ASPECTS <7 mean volume = 64.6cc, Std 42.5cc, ≥7 mean volume = 22.7cc, SD 19.4cc (Figure 5).

### 3.6. Measured Infarct Volume Correlated with Decision to Treat

The clinical decision to treat with IV-tPA is made based on NCCT imaging and clinical history, with consideration for infarct size and acuity. Only the initial images for each case were used (29 cases, one case with mechanical thrombectomy not included). IV-tPA was administered in 9 cases, FSL mean measured infarct volume 25.7cc, SD 13.9cc, and the decision was made not to treat in 20 cases (mean infarct volume 67.5cc, SD 39.4cc). The measured infarct volumes of the two groups were significantly different (Student's t-test p < 0.005). All cases where treatment was elected measured at less than 50cc. For ITK treated mean volume 16.9cc, SD 19.6cc and untreated 51.5cc, SD 32.7cc. The measured infarct volumes of the two groups were significantly different (Student's t-test p=0.007). All cases where treatment was elected measured less than 52cc.

## 4. Discussion

Knowledge of the size of an acute infarct is important in clinical decision-making. The risk of hemorrhage and poor functional outcomes is increased with larger strokes, and thrombolysis carries a greater risk of hemorrhage in larger and more mature strokes [21]. NCCT remains the first-line modality, and more advanced imaging may not be available or cost effective in the emergency setting. The goal of this study is to develop a digital image analysis pipeline for estimating the size of an acute ischemic stroke from NC head CT images. The proposed method is based on the idea that acute ischemic stroke appears as an image intensity inhomogeneity on noncontrast head CT images, and therefore image intensity inhomogeneity software might be used to characterize acute ischemic stroke.

Image intensity inhomogeneity or “bias field” has been most extensively studied in MRI, and various strategies for bias field correction have been proposed [22, 23]. Image intensity correction has received considerably less attention in CT imaging. We had previously observed that a preprocessing algorithm from FSL (FSL FAST) can be applied to brain CT images, to identify and reduce subtle but persistent image inhomogeneities, largely arising from beam hardening and “cupping artifact” caused by the skull, enabling direct image segmentation [20]. The bias field reduction algorithm in FSL will identify and reduce regional inhomogeneities in signal intensity and will generate a “corrected” image (referred to as the “restored” image in FSL). If the “restored” image is subtracted from the input image, the size and magnitude of the image intensity inhomogeneity can be determined. We propose that an image analysis algorithm can be generated where IIC mapping and intensity thresholding are used to identify acute infarcts and to measure the volume of an acute infarct. Such a capability would be quantitative and objective and amenable to automation.

Infarct volume seen on noncontrast CT images increases over time as infarct volume measured from noncontrast CT is a function of both the infarct size and the infarct attenuation at the time of the measurement. Digitally created synthetic infarcts afford an opportunity to investigate the dependence of volumetric estimations on each of these variables. We investigated the dependence of two different IIC algorithms on infarct size and attenuation. Figure 3 illustrates the relationship between measured infarct volume and true infarct volume, evidencing several points: (1) Measured infarct volume accuracy improves with increasing infarct size and attenuation. (2) The relationship between measured and true volumes is nearly linear but the slope (and coefficient of determination) increases as the stroke radiodensity decreases (or change in radiodensity increases). (3) Small infarcts with lesser density change are measurable, though a baseline volume due to inherent asymmetry introduces a nonzero calculated volume (16cc for FSL, 3cc for ITK) when the true volume is zero. These nonzero baseline values of stroke volume and radiodensity are likely caused by asymmetries of the brain and skull images and reduce the accuracy of smaller measurements. Together, Figures 2 and 3 and Table 1 show that, in the ideal case, the digital method is capable of detecting infarcts below the 2-4HU decrease regarded as the limit of what the human eye can detect [24, 25]. FSL IIC values were also more highly correlated with the ASPECTS score.

Historically, studies have shown that the risk of hemorrhage increases with increasing stroke size, and this risk is increased in the presence of IV-tPA [1, 3, 4, 21], which has led to dichotomized guidelines for IV-tPA administration based on estimations of stroke size [1, 7, 26]. Although current guidelines acknowledge that data are limited and do not advocate withholding IV-tPA in acute stroke based on estimations of stroke size alone [27, 28], objective, quantitative volumetric measures may serve to improve risk stratification in the future. It is important to appreciate however that the accuracies of ASPECTS scores or other methods of volumetric estimation are not verified against a “ground truth,” and the exact infarct volume is probably not as important as the consistency and reproducibility of the measure, which argues for the value of an automated method. The method we describe is based on open source software and requires a minimum amount of off-line processing. The method is sensitive to very small changes in radiodensity and generates an illustrative map of the detected changes, which can then be analyzed to estimate the infarct volume.

Validating the accuracy of the method in clinical stroke proves challenging as there is no “gold standard” for estimating stroke volume on NCCT in the most acute stages, before the margins of the stroke are delimited. Ideally, concurrent MRI with diffusion weighted imaging may offer such a gold standard, but such data is rarely available and time delays between NCCT and MRI limit the value of such comparisons. We use several different approaches to evidence validity of the infarct volume calculations. Firstly, we investigated two different IIC software programs to show that the idea was valid across platforms. Second, synthetic, digitally created hypodensities were used to show strong correlation between the measured volume and the known volume of the hypodensity. Thirdly, clinical cases were investigated to show correlation with clinical parameters of ASPECTS scoring, NIHSS, and decision to treat with IV-tPA. These studies show that both IIC algorithms have the capability to identify and measure the volumes of subtle hypodensities of an acute infarct. The algorithms prove nonidentical in their measures, and though measures were highly correlated with each other, the segmentation-derived FSL algorithm proved more robust for estimating stroke volume on NCCT by many of the measured parameters. When possible we measured infarct volumes manually to find a strong correlation between manual measures and volumes calculated using the automated IIC methodology.

Clinical head CT imaging is subject to a number of artifacts, largely caused by the skull, which create image intensity inhomogeneities that limit routine image interpretation and also limit the quantitative method outlined here, resulting in a “false positive” baseline in our investigation. In addition to acting as a clinical tool, image intensity inhomogeneity analysis can provide information which may prove useful in identifying, characterizing, and rectifying inhomogeneities on noncontrast clinical head CT images, an improvement which would lead to greater sensitivity for identifying early ischemic changes.

## 5. Conclusions

This is a pilot investigation into the use of IIC and digital image analysis for the characterization of acute infarct on NC head CT. The method enables objective estimation of infarct size, in some cases even before the infarct is clearly visible on the routine images, providing information which may prove useful in stroke management.

## Figures and Tables

**Table 1:** Ratios of synthetic infarct volume and the corresponding measured volume as a function of changes in volume (three volumes at left) and different radiodensities (four densities across the top of the columns) for the two different IIC algorithms (FSL and ITK). The numbers correspond to Figure 3.

	30%		20%		10%		5%	
	FSL	ITK	FSL	ITK	FSL	ITK	FSL	ITK
122cc	92.2%	69.4%	81.2%	53.1%	53%	31.9%	34.9%	19.6%
45cc	128.1%	114.3%	117.6%	92.0%	86.5%	59.0%	64.0%	39.0%
17cc	210%	176.7%	201.8%	148.5%	160.4%	102.3%	131.6%	66.4%

## Data Availability

The data used to support the findings of this study are available from the corresponding author upon request.

## Supplementary Materials

Supplementary Materials Table 2: demographics of acute stroke cases.
