# Supplementary material for: Automated Estimation of Acute Infarct Volume from Noncontrast Head CT Using Image Intensity Inhomogeneity Correction
Source: Int J Biomed Imaging. 2019 Aug 21;2019:1720270. doi: 10.1155/2019/1720270 (PMC6719274; doi:10.1155/2019/1720270)
Supplement: Supplementary Materials — Table 2: demographics of acute stroke cases. [file 1720270.f1.docx]

|  | **Age, Y** | **Sex** | **Side** | **Occlusion** | **Lesion age**  **at ED** | **Rt-PA** | **Time to CT** | **Time to MRI** | **NIHSS** | **Est Vol fsl (cc)** |
| --- | --- | --- | --- | --- | --- | --- | --- | --- | --- | --- |
| **1** | 69 | M | R | MCA | unknown | no | unknown | 27 hrs | 18 | 71.5 |
| **2** | 88 | F | L | MCA | LKWT  27 hrs | no | <27 hrs | 8 hrs | - | 70 |
|  |  |  |  |  |  |  | +6.5 hrs |  |  | 82.9 |
| **3** | 84 | M | R | MCA | 2 hrs (xfer) | yes | 5 hrs | No MRI  CT 3 d | 34 | 78.7 |
|  |  |  |  |  |  |  | +14hrs |  |  | 144.2 |
| **4** | 69 | F | L | MCA | ~11 hrs | no | ~11 hrs | 3 hrs | 20 | 87.3 |
| **5** | 83 | M | L | M2/M3 | <3 hrs | yes | <3 hrs | 48 hrs | 6 | 38.2 |
| **6*** | 62 | M | L | MCA | unknown | no | unknown | 4 d |  | 57.8 |
| **7** | 57 | M | R | MCA M2 | unknown | no | unknown | 9 hrs | 10 | 37.8 |
| **8** | 84 | F | L | MCA | ~7 hrs | no | ~7hrs | 6 hrs | 25 | 67.8 |
| **9*** | 48 | F | L | MCA | <1hr (xfer) | yes | 7.5 hrs | 24 hrs |  | 19.2 |
| **10*** | 83 | M | R | MCA | ~1 hr | yes | ~1hr | 18 hrs | 31 | 29.8 |
| **11** | 77 | M | L | MCA | <2 hrs | no | <7hrs | 28 hrs | 34 | 177.5 |
| **12*** | 61 | M | L | MCA | < 1hr | yes | <1hr |  |  | .65 |
|  |  |  |  |  |  |  | 25 hrs | 3 d | 3 | 6.6 |
| **13** | 82 | F | R | MCA | 1 hr | yes | 1.25 hrs |  |  | 24.2 |
|  |  |  |  |  |  |  | 2.5 hrs |  | 21 | 41.6 |
|  |  |  |  |  |  |  | 26 hrs | 36 hrs |  | 41.1 |
| **14** | 57 | M | R | MCA | unknown | no | unknown |  |  | 113.6 |
|  |  |  |  |  |  |  | +3.75 hrs | 12 hrs |  | 26.1 |
| **15** | 67 | M | L | MCA | LKWT 3d | no | <3d |  | 9 | 39 |
|  |  |  |  |  | + 40 hrs |  | +40 hrs | 40 hrs |  | 80.4 |
| **16** | 66 | F | L | MCA M1 | 3.5 hrs | yes | 5.5 hrs | 40 hrs | 31 | 64.5 |
| **17** | 66 | F | R | MCA | 13 hrs | no | unknown | 17 hrs | 6 | 59.4 |
| **18** | 46 | M | L | MCA | 8 hrs | yes | 14 hrs |  | 15 | 6.3 |
|  |  |  |  |  |  |  | 30 hrs | 2 d |  | 36.7 |
| **19** | 83 | F | L | MCA | LKWT  23 hrs | no | 14.5 hrs | 40 hrs | 14 | 72.5 |
| **20** | 76 | M | R | MCA M2 | unknown | no | unknown | 28.5hrs |  | 56.4 |
| **21**** | 41 | M | R | MCA M1 | 1.3 hrs | yes | 2.5 hrs | 33 hrs | 19 | 12 |
|  |  |  |  |  |  |  | 5 hrs |  |  | 81.4 |
|  |  |  |  |  |  |  | 28 hrs |  |  | 117.9 |
| **22** | 79 | M | R | MCA M1 | unknown | no | unknown | No MRI CT 8d | 18 | 105 |
| **23** | 64 | M | R | MCA M1 | 1hr | yes | 1hr | 46hrs | 12 | 2 |
| **24*** | 70 | M | L | ICA | 1hr | yes | 1hr | 20hrs |  | 44.8 |
|  |  |  |  |  |  |  | 7hrs |  |  | 286.4 |
| **25** | 65 | M | L | MCA M1 | 1.5hrs | no | 2hrs | 9hrs | 21 | 54.6 |
| **26** | 101 | F | L | ICA | 4hrs | no | 4hrs | 15hrs | 28 | 124 |
| **27** | 65 | F | R | ICA | 6hrs | no | 3.5hrs | 24hrs | 9 | 34 |
| **28** | 85 | M | L | ICA | 2.5hrs | embolect | 2.5hrs | 16 hrs | 23 | 28.1 |
|  |  |  |  |  |  |  | 4.5 hrs |  |  | 35.6 |
| **29** | 55 | F | L | ICA | LKWT  36hrs | no | unknown | 3d |  | 67.9 |
| **30*** | 46 | M | R | ICA | unknown | no | unknown | 21.5hrs |  | 71.5 |

**Table 2: Demographics of acute stroke cases.**

LKWT – last known well time

*Infarct not appreciated at interpretation of the initial study
